# Association Between Self-Reported Health and Dementia Symptoms Among People With Dementia and Their Caregivers

**DOI:** 10.1093/geroni/igaa057.232

**Published:** 2020-12-16

**Authors:** Eric Jutkowitz, Mauricio Lopez Mendez, Rowan Iskandar, Laura Pizzi

**Affiliations:** 1 Brown University, Providence, Rhode Island, United States; 2 Brown University, Providence, United States; 3 Rutgers Ernest Mario School of Pharmacy, Piscataway, New Jersey, United States

## Abstract

People living with dementia (PwD) can often reliably self-report their health; yet, there are limited data on their and their primary caregiver’s self-reported overall health (excellent, very good, good, fair, and poor). We used data from the Aging, Demographics, and Memory Study (2001-2009) to quantify the association between PwD’s cognitive impairment (Mini-mental State Exam), physical limitations (scale [0-10] of activities of daily living), and behaviors (scale [0-12] of behavioral symptoms on the Neuropsychiatric Inventory Questionnaire) and self-reported health. We estimated two ordered logistic regressions estimating: 1) PwD’s self-reported health (analyzed n=308); 2) primary caregiver’s self-reported health (analyzed n=135; 173 PwD did not have primary caregiver in the survey). We controlled for the PwD demographics, chronic conditions, and if they lived in the community. The regression estimating caregiver’s self-reported health also controlled for the caregiver’s relationship to the PwD, and whether the caregiver lived with the PwD. PwD’s self-reported health was lower (4% excellent; 16% very good; 22% good; 30% fair; 30% poor) than caregivers (14% excellent; 27% very good; 32% good; 23% fair; 4% poor). For PwD, one-additional physical limitation, but not cognition or behavior, was associated with 1.15 (95%CI: 1.01,1.30) times greater odds of self-reporting poor health compared to all other categories. For caregivers, one-additional behavior, but not cognition or physical limitations, was associated with 1.17 (95%CI: 1.01,1.37) times greater odds of self-reporting poor health. For PwD, interventions targeting physical limitations may increase self-reported health, but for caregivers, interventions targeting behavioral symptoms may increase self-reported health.

